# Genomic Characterization of Drug-Resistant *Mycobacterium tuberculosis* L2/Beijing Isolates from Astana, Kazakhstan

**DOI:** 10.3390/antibiotics12101523

**Published:** 2023-10-10

**Authors:** Dana Auganova, Sabina Atavliyeva, Asylulan Amirgazin, Akmaral Akisheva, Anna Tsepke, Pavel Tarlykov

**Affiliations:** 1 National Center for Biotechnology, Astana 010000, Kazakhstanamirgazin@biocenter.kz (A.A.); 2 City Center for Phthisiopulmonology of the Akimat of Astana, Astana 010000, Kazakhstan

**Keywords:** *Mycobacterium tuberculosis*, drug resistance, compensatory mutations, Kazakhstan, whole-genome sequencing, genetic diversity

## Abstract

Kazakhstan ranks among the countries with the highest number of MDR-TB patients per 100,000 population worldwide. The successful transmission of local MDR strains of *Mycobacterium tuberculosis* (Mtb) poses a significant threat to disease control. In this study, we employed whole-genome sequencing to examine drug resistance, compensatory mutations, population structure, and transmission patterns in a sample of 24 clinical isolates of L2/Beijing Mtb collected in Astana, Kazakhstan between 2021 and 2022. The genotypic prediction of Mtb susceptibility to anti-TB agents was consistent with the phenotypic susceptibility, except for bedaquiline. An analysis of resistance-associated genes characterized most of the isolates as pre-extensively drug-resistant tuberculosis (pre-XDR-TB) (n = 15; 62.5%). The phylogenetic analysis grouped the isolates into four transmission clusters; the dominant cluster was assigned to the “aggressive” Central Asia outbreak (CAO) clade of L2/Beijing (n = 15; 62.5%). Thirteen mutations with putative compensatory effects were observed exclusively in Mtb isolates containing the *rpoB* S450L mutation. The putative compensatory mutations had a stabilizing effect on RpoABC protein stability and dynamics. The high prevalence of the CAO clade in the population structure of Mtb may explain the rapid spread of MDR-TB in Kazakhstan.

## 1. Introduction

Tuberculosis (TB), which is caused by the *Mycobacterium tuberculosis* complex (MTBC), is a global public health concern. The most clinically relevant species of MTBC is *Mycobacterium tuberculosis* (Mtb). According to a recent WHO report, approximately 6.4 million newly diagnosed cases of TB and 1.6 million deaths were registered in 2021 versus 5.8 million cases and 1.5 million deaths in 2020 [1]. The TB incidence in 2021 rose by 4.5% compared to that in 2020, from 10.1 million to 10.6 million. The consequences of the COVID-19 pandemic may have influenced both TB incidence and mortality. This is also the first time in more than a decade that TB mortality has increased [2]. In addition, about 23% of the global population is infected with latent TB (LTBI) [3]. According to the data, 5–10% of LTBI cases could potentially turn into an active form of TB during the patient’s lifetime [4,5]. Given the risks involved, the epidemiology of TB plays a significant role in the prediction of its transmission.

The emergence of TB’s resistance to anti-TB drugs is a significant challenge. The burden of drug-resistant TB (DR-TB) is estimated to have increased between 2020 and 2021, with an estimated 450,000 new cases of rifampicin-resistant TB reported in 2021 [1]. According to the latest TB report by the WHO, Kazakhstan is among the 30 countries with the highest rates of multi-drug-resistant TB (MDR-TB)/rifampicin-resistant TB (RR-TB). The total incidence rate of MDR/RR-TB was 29 cases per 100,000 in 2021 [6]. Kazakhstan has witnessed a decline in both the incidence and rate of mortality from TB during the past decade, but the incidence of multidrug-resistant TB remains alarmingly high. In 2021, MDR-TB was detected in 34% of newly diagnosed TB patients and 49% of retreatment cases [6].

Currently, local bacteriology laboratories in Kazakhstan utilize Xpert MTB/RIF, a method based on real-time PCR developed by Cepheid in the UK, to rapidly diagnose TB disease and drug resistance. This diagnostic tool is known for its speed and effectiveness. However, it does have several limitations, such as its limited ability to detect drug resistance. The assay provides information about rifampicin resistance, but it does not provide comprehensive drug resistance data for novel TB drugs such as delamanid and pretomanid. Whole genome sequencing (WGS) has proven to be invaluable for overcoming these limitations. WGS enables complete screening of the genome sequence of an Mtb isolate, thereby providing comprehensive information on drug resistance and other genetic characteristics [7]. The use of WGS in TB research in Kazakhstan has been limited to a handful of studies. A PubMed search using the terms “whole-genome sequencing”, “Kazakhstan”, and “tuberculosis” found only six publications, describing a total of 97 Sequence Read Archive (SRA) whole-genome sequences of local Mtb isolates. This suggests that the use of WGS in TB research and surveillance is not yet widespread in Kazakhstan [8,9,10,11,12,13].

The acquisition of drug resistance in Mtb primarily involves genetic mutations in specific genes associated with drug targets or drug activation (*rpoB*, *katG*, *inhA*, *gyrA*, *pncA*, etc.). Mutations in the *rpoB* gene, which encodes the beta subunit of RNA polymerase (RNAP), are the most common cause of resistance to rifampicin, a key first-line TB drug [14,15,16]. RNAP is essential for transcription in the bacterium. This process is vital for gene expression, the synthesis of RNA molecules, and the regulation of various cellular processes, including those related to the ability of mycobacteria to cause tuberculosis infections in humans. Furthermore, mutations in *rpoABC* targeting RNAP can also affect bacterial fitness and virulence [17].

The aim of our study was to characterize the population structure, drug resistance, and compensatory mutations of a set of 24 L2/Beijing isolates from the capital city of Kazakhstan via WGS in order to provide a deeper insight into the drug resistance and population structure of local Mtb strains.

## 2. Results

### 2.1. Sample Selection and Sequencing

Starting from 29 candidate Mtb isolates collected in Astana, we excluded 5 non-Beijing isolates using a real-time PCR assay specifically for L2/Beijing isolates (Figure S1). This assay targets the *dnaA-dnaN*::IS*6110* region of the L2/Beijing family of Mtb [18]. The presence of L2/Beijing mycobacterial DNA was confirmed by an exponential increase in the FAM channel signal, while other non-Beijing genotypes produced an increased signal in the HEX channel. As a result, 24 clinical isolates with L2/Beijing genotypes were selected for WGS. For the study, 24 TB patients (10 women and 14 men aged 25–61 years) were recruited at the City Center of Phthisiopulmonology in the Akimat of Astana in 2021–2022 (Table 1). The majority of the patients were diagnosed with infiltrative pulmonary TB (n = 20; 83.3%), while the others were diagnosed with miliary TB (n = 2; 8.3%) and fibrocystic cavernous TB (n = 2; 8.3%). Twenty-four sequencing libraries were prepared and paired-end sequenced. The paired-end reads were successfully generated and mapped to the *M. tuberculosis* H37Rv genome. The mapping showed an average read depth of 119× and a mean genome coverage of 98.9%.

### 2.2. Population Structure

The population structures of the L2/Beijing isolates were characterized using five genotyping methods: (1) in silico spoligotyping [19], (2) assignment of regions of deletion (RD) [20], (3) 62 SNP barcoding according to Coll et al. [21], (4) classification according to Merker et al. [22], (5) and SNP barcoding for Mtb lineage 2 according to Thawornwattana et al. [23] (Table 2). In silico spoligotyping led to an identical spoligotype for all the studied isolates: octal code 000000000003771 (SIT 1 or SIT1/Beijing). In addition, an identical set of RDs (RD105, RD207, RD181), which is commonly referred to as the Beijing genotype, was assigned to all samples. Moreover, a phylogenetically informative SNP in position 797736 (C/T) specific to lineage 2.2.1 was identified in all Mtb isolates. A more detailed population structure of the Mtb collection was revealed in the L2/Beijing subgroup classification by Merker et al. and Thawornwattana et al. [22,23]. According to the published SNP barcodes, the majority of studied L2/Beijing isolates (n = 15; 62.5%) were assigned to the Central Asia outbreak (CAO) sublineage, also known as L2.2.M4.9.1, according to Thawornwattana et al.’s classification. Other sublineages were represented by Central Asia (CA) L2.2.M4.9 (n = 5; 20.8%), Europe/Russia W148 outbreak L2.2.M4.5 (n = 3; 12.5%), and Clade A L2.2.M4.9.2 (n = 1; 4.2%).

### 2.3. Drug Resistance-Associated Mutations

The genotypic prediction of drug resistance was performed for the mycobacterial isolates (Table 3). The majority of the studied L2/Beijing isolates were RIF-resistant (n = 21; 87.5%), bearing S450L (19/24) or H445Y (2/24) mutations in the *rpoB* gene. Amino acid changes at codons 450 and 445 in the *rpoB* gene correspond to codons 531 and 526, respectively, in the old *Escherichia coli* nomenclature. The same prevalence (n = 21; 87.5%) of INH resistance (*katG* gene S315T mutation), STM resistance (*rpsL* K43R mutation), and EMB resistance (*embCAB* Operon mutations) was observed in the studied samples. The most prevalent mutations responsible for EMB resistance were *embB* M306V (15/24) and *embA* -16C>T (3/24). Pyrazinamide resistance was attributed to *pncA* gene mutations (n = 9; 37.5%). *pncA* D63A, W68R, and V139A mutations were detected in two Mtb isolates, while mutations L85R, I133T, and 287delA were detected only once. Fluoroquinolone resistance was associated with gyrase mutations, including mutations in the *gyrA* and *gyrB* genes (n = 16; 66.7%). The most prevalent mutations conferring resistance to OFX were *gyrA* D94G (5/24), A90V (4/24), D94N (2/24), D94A (1/24), and S91P (1/24). In addition, the *gyrB* gene mutation T500P was found in two isolates, and *gyrB* D461H was detected once. Drug resistance to the anti-TB drugs KAN, AMK, and CAP was associated with mutations in the *rrs*, *eis*, and *tlyA* genes (n = 5; 20.8%). An 1401A/G mutation in the *rrs* gene was found in two isolates (2/24); the mutation -37G>T in the *eis* gene was detected with the same frequency (2/24), while the 395dupA mutation in the *tlyA* gene was encountered only once (1/24). Ethionamide-associated mutations in the *inhA* and ethA genes were present in two Mtb isolates (2/24): the S94A mutation in the *inhA* gene (1/24) and the -7T>C mutation in the *ethA* gene (1/24). BDQ- and CFZ-associated resistance was driven by *Rv0678* gene mutations (n = 4; 16.7%), including G66E and S68G mutations and 193delG deletion.

As a result, the genomic drug susceptibility testing characterized Mtb isolates as sensitive (n = 2; 8.3%), other (n = 1; 4.2%), MDR (n = 5; 20.8%), Pre-XDR (n = 15; 62.5%), and XDR (n = 1; 4.2%) (Table 1 and Table 3). Heteroresistance was not detected in the studied isolates, since no mixed calls with less than 95% of the read depth were assigned in the alleles associated with drug resistance.

### 2.4. Comparison of Phenotypic Drug Susceptibility Testing and Genotypic Prediction

Phenotypic DST with respect to the anti-Tb drugs was conducted in the bacteriology lab using the MGIT 960 system with a sample of 24 local Mtb isolates (Table S1). The results of the phenotypic DST were found to be consistent with the drug resistance profiles obtained from genomic data, except for BDQ. We found phenotype–genotype discrepancies for two isolates (#6242 and #6466) with a phenotype of BDQ resistance (Table 1, Table S1) that was not confirmed with genotypic prediction. The other four isolates phenotypically resistant to BDQ harbored mutations in the *Rv0678* gene (G66E, S68G, and 193delG) conferring resistance, as described by Sonnenkalb et al. [24]. Furthermore, the deletion 193delG in the *Rv0678* gene conferred cross-resistance between BDQ and CFZ in isolate #1561; this cross-resistance was confirmed phenotypically.

### 2.5. Phylogenetic Analysis

**Figure 1:**
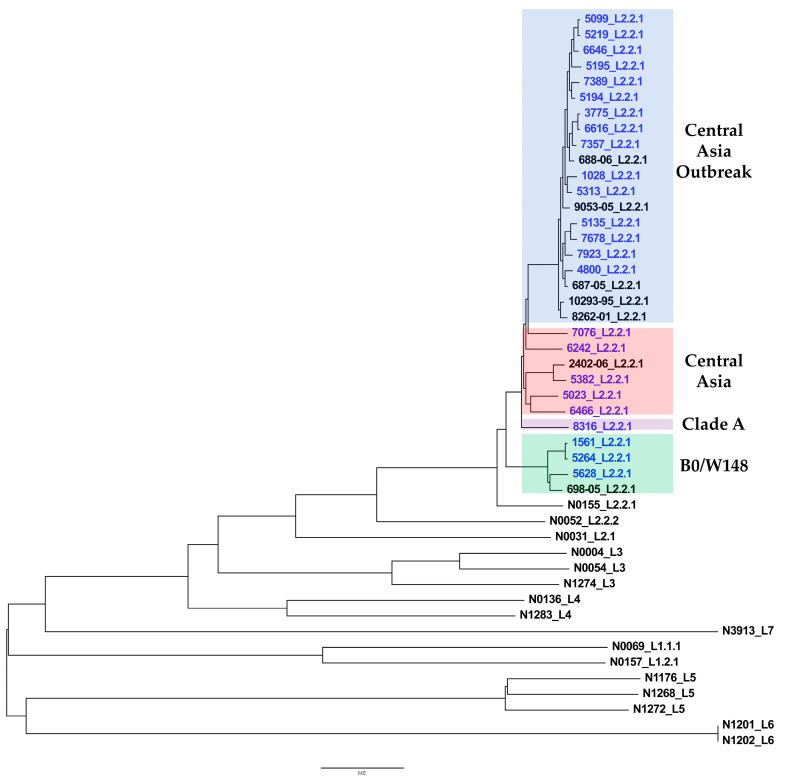
Maximum likelihood tree (general time reversible model of nucleotide substitution, 1000 resamplings, and Gamma20 likelihood optimization) of 47 MTBC whole-genome sequences, including 24 sequences from the current study (in blue), 7 sequences of L2/Beijing isolates studied by Merker et al., and 16 reference sequences of MTBC clinical strains published by Borrell et al. [25,26]. The consensus tree was rooted with a “midpoint root” option, and the nodes were arranged in increasing order. The branch lengths are proportional to nucleotide substitutions. The scale bar indicates substitutions per site. The sublineages are labeled according to Coll et al. [21]. The population structure of the samples from Astana, Kazakhstan, is represented by four genetic clades: Central Asia outbreak, Central Asia, Clade A, and Europe/Russia W148 outbreak.

Maximum-likelihood phylogenetic analysis was conducted. A phylogenetic tree was constructed based on the overall SNPs extracted from 47 MTBC genomic DNA sequences, including WGS data from this study and a study by Merker et al. (Figure 1 and Table S1) [25]. Phylogenetic analysis grouped the genomes described in this study into four genetic clusters corresponding to the L2/Beijing subgroup classification described earlier [22]. Genetic clusters are represented by CAO, CA, Clade A, and Europe/Russia W148 outbreak sublineages.

The calculated pairwise distance matrix shows the genetic distances between samples (Table S2). Smaller distances indicate closely related isolates. A maximum distance of 12 SNPs between Mtb isolates was considered to indicate a possible epidemiological link between TB cases [27]. According to this criterion, three pairs of isolates were closely related and possibly linked epidemiologically (#5264–1561, #6616–3775, and #5219–5099).

### 2.6. Compensatory Mutations

**Figure 2:**
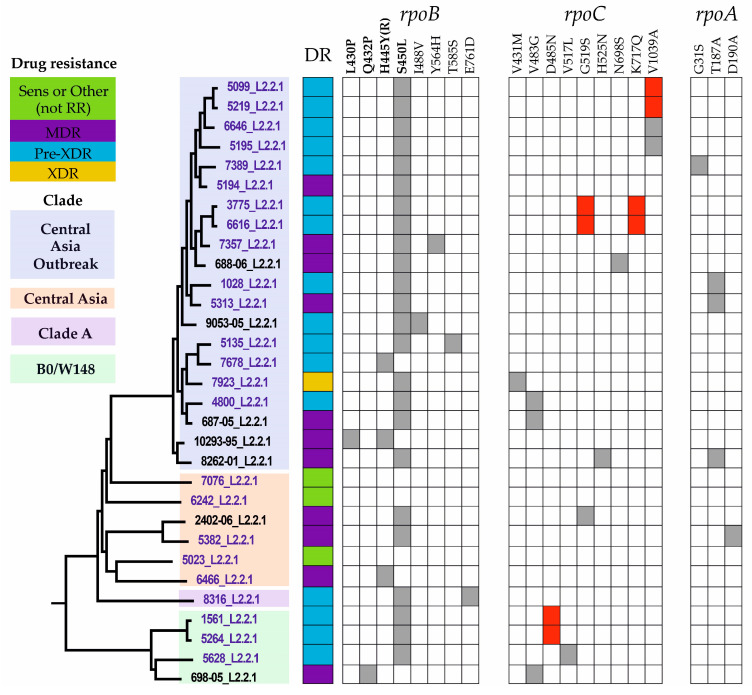
Population structure, drug resistance, and compensatory mutations of *M. tuberculosis* L2/Beijing isolates from Astana, Kazakhstan. Maximum likelihood tree (general time reversible model of nucleotide substitution, 1000 resamplings, and Gamma20 likelihood optimization) of Mtb isolates from the current study (in blue) and L2/Beijing isolates from a study by Merker et al. [25]. For convenience, only a cluster of L2.2.1 isolates is shown. Drug resistance of the Mtb isolates was classified as sensitive or other, excluding rifampicin-resistance (Sens or Other (not RR)), MDR, Pre-XDR, or XDR. Drug resistance-conferring mutations in *rpoB* are in boldface. Compensatory mutations in epidemiologically linked pairs of Mtb isolates (#5264–1561, #6616–3775, and #5219–5099) are highlighted in red. The population structure of the studied L2/Beijing isolates is represented by four genetic clades: Central Asia outbreak, Central Asia, Clade A, and Europe/Russia W148 outbreak.

In the context of this study, we only considered a variant in *rpoA*, *rpoB*, or *rpoC* as a putative compensatory variant when it co-occurred with an RIF resistance (RR)-conferring mutation. We excluded known phylogenetic variants such as *rpoC* E1092D, silent mutations, and RR-determining regions (*rpoB* codon 425-452). As a result, a total of 13 putative compensatory mutations were detected in the studied L2/Beijing isolates, 7 in *rpoC* and 3 each in *rpoB* and *rpoA* (Table 4 and Figure 2). Two putative compensatory mutations were novel: *rpoC* mutation K717Q and *rpoB* mutation T585S. All putative compensatory mutations were linked to a single RR *rpoB* S450L mutation, while another one, H445Y, was not accompanied by any non-synonymous variants in *rpoA*, *rpoB*, or *rpoC*. The most frequent compensatory variant, *rpoC* V1039A, was found in four isolates. Two of the four (#5219–5099) were linked epidemiologically, as inferred from the pairwise distance matrix (Table S2). The observed compensatory mutations may have arisen in response to the fitness costs associated with primary resistance mutations by affecting the growth and fitness of Mtb. However, we could not conduct direct measurements of the growth rate of the resistant strain with compensatory mutations compared to the resistant strain that lacked mutations to confirm its connection to phenotype due to laboratory biosafety restrictions.

**Figure 3:**
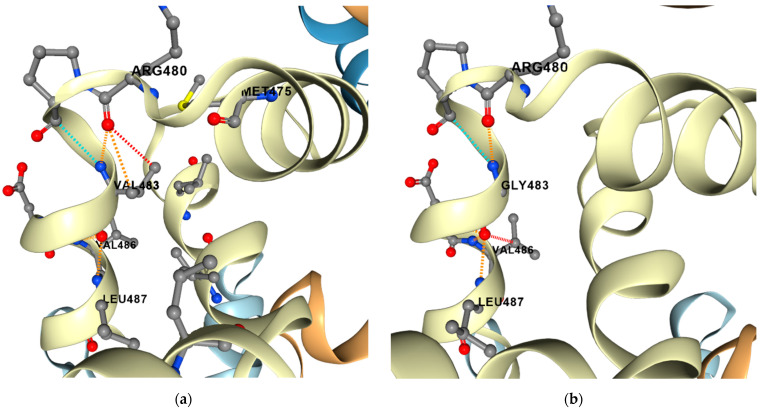
Three-dimensional representation of compensatory mutations on the crystal structure of Mtb DNA-directed RNA polymerase (RNAP) subunit beta (PDB: 6c04). (**a**) Wild-type (WT); (**b**) mutant V483G. Wild-type and mutant residues, represented as sticks alongside surrounding residues, are involved in different types of interactions (orange: polar; red: hydrogen bond; blue: Van der Waals force). Val to Gly transition in RNAP subunit beta led to a potentially destabilizing negative ΔΔG value as predicted by DynaMut (http://biosig.unimelb.edu.au/dynamut/ accessed on 3 July 2023).

The impact of compensatory variants on protein stability and dynamics was assessed in silico by calculating the changes in folding free energy (ΔΔG, kcal/mol) and vibrational entropy (ΔΔS_Vib_, kcal/mol) between the wild type and mutant. It was found that 11 out of 13 compensatory variants had negative values for changes in vibrational entropy, indicating a decrease in molecule flexibility; a total of 12 out of 13 putative compensatory mutations had positive ΔΔG values, leading to a potentially stabilizing effect on DNA-directed RNA polymerase (RNAP) subunits (Table 1). The only exception was the *rpoC* V483G mutation (Figure 3) in CAO isolate #4800, which had a negative ΔΔG value, with a potentially destabilizing effect on the subunit structure. Despite the negative ΔΔG value, this compensatory variant appeared to have evolved convergently in the Mtb isolate from genetic clade B0/W148 (#698-05, Uzbekistan). The *rpoC* V483G compensatory mutation led to the rigidification of the RNAP structure (decreased flexibility shown in blue), with a negative vibrational entropy value, compared to wild-type RNAP (Figure S2a).

Another convergently acquired compensatory variant is *rpoC* G519S, which appears to be genetically distant from both CAO (#3775 and 6616, this study) and CA (#2402-06, Uzbekistan) clades. Interestingly, both convergently evolved compensatory mutations, V483G and G519S, are located in the same region of subunit beta’, encoded by *rpoC* (Figure S2b).

## 3. Discussion

Only a handful of regional studies have used the WGS approach to characterize the MTBC population in Kazakhstan. The sample size in these studies usually does not exceed 10 isolates due to budget constraints and/or lack of funding [8,9,11,12,28,29]. As a result, WGS technology has not been widely adopted in Kazakhstan in clinical or other settings. However, WGS of MTBC has rapidly progressed in developed countries from a research tool to a clinical or public health application [7,30,31]. In the current study, WGS sequences of 24 clinical isolates of L2/Beijing Mtb collected in Astana, Kazakhstan, were characterized to examine drug resistance, compensatory mutations, population structure, and possible transmission patterns.

Our data show that the genotypic prediction of Mtb susceptibility to anti-TB agents was consistent with the phenotypic susceptibility. Phenotypic and genomic DST data of L2/Beijing isolates collected in Astana revealed that the majority of the studied Mtb isolates were resistant to RIF and INH (n = 21; 87.5%) (Table 3). However, the numbers do not necessarily represent actual resistance prevalence in the general population of Astana, since new TB cases (n = 13; 54.2%) were included in the sampling along with relapse (n = 9; 37.5%) and retreatment cases (n = 2; 8.3%) (Table 1). As a result, the sampling was biased in favor of recurrent TB. Notably, an earlier study of 700 Mtb clinical isolates from 12 regions in Kazakhstan in 2010–2015, using the TB-SPRINT and TB-SNPID hybridization methods, revealed that 60% of all samples were resistant to RIF and INH [32].

Phenotype- and genotype-based DST data were consistent, except for one group A drug, BDQ. The results of phenotypic DST identified six isolates with BDQ resistance (n = 6; 25%). To date, high-confidence BDQ-associated mutations have not been reported in the WHO catalogue of Mtb mutations. Thus, a list of BDQ-associated mutations by Sonnenkalb et al. was used to evaluate the detected genetic variants [24,33]. Typically, most strains phenotypically resistant to BDQ show mutations in the *Rv0678* gene [34]. As a result, four out of six isolates that were phenotypically resistant to BDQ were found to harbor mutations in the transcriptional repressor *Rv0678*, which regulates the MmpS5–MmpL5 efflux pump. In the current study, a loss-of-function frameshift mutation 193delG in the *Rv0678* gene was found in two isolates. This deletion confers cross-resistance between BDQ and CFZ in one isolate, as described by Vargas et al. [35]. Importantly, BDQ has been considered a core drug for the treatment of MDR-TB in Kazakhstan since 2018 [36]. Thus, the acquisition of additional resistance to BDQ in local strains may contribute to the emergence of XDR-TB, which is even more difficult to treat and control than MDR-TB.

An analysis of resistance-associated genes characterized most of the studied isolates as pre-extensively drug-resistant TB (pre-XDR-TB) (n = 15; 62.5%). These isolates fulfill the new WHO definition of pre-XDR-TB; i.e., they are multi-drug and rifampin-resistant as well as resistant to any fluoroquinolone [37]. Moreover, a single isolate (#7923) was characterized as XDR-TB (n = 1; 4.2%). Both pre-XDR-TB and XDR-TB are critical stages in the progression of drug-resistant TB, which poses a formidable public health challenge. Both forms lead to increased treatment costs, compromised therapeutic outcomes, and the potential for widespread transmission. The extensive transmission of drug-resistant strains could potentially lead to community outbreaks. To check this possibility, we analyzed transmission patterns by considering a maximum distance of 12 SNPs between the Mtb isolates as a possible epidemiological link between TB cases [28]. Although all samples were collected in the same region (Astana, Kazakhstan), only three pairs of isolates were closely related to each other and possibly linked epidemiologically, as revealed from the pairwise distance matrix (#5264–1561, #6616–3775, and #5219–5099) (Figure S2). In addition, each of these Mtb isolate pairs were characterized as pre-XDR-TB and shared an identical set of putative compensatory mutations (Figure 2, Table S1).

In a country-wide study, Klotoe et al. reported that nearly 80% of 700 local isolates were characterized as L2/Beijing [32]. Notably, the L2/Beijing family is known for its high virulence and accumulation of drug resistance-conferring mutations, mainly associated with an MDR genotype [22,25]. According to a classification by Thawornwattana et al., L2 Modern Beijing is classified into six main clades, denoted as L2.2.M1–L2.2.M6. In Kazakhstan, a dominant genotype of Modern Beijing Mtb is L2.2.M4, comprising several sublineages: Europe/Russia W148 outbreak (L2.2.M4.5), CA (L2.2.M4.9), CAO (L2.2.M4.9.1), and Clade A (L2.2.M4.9.2) [23].

Recently, Merker et al. reported the dominance of a CAO transmission cluster in neighboring Uzbekistan [25]. Uzbekistani samples were studied using the WGS approach to determine the evolutionary history and clonal expansion of the dominant CAO clade. It was concluded that the CAO clade had driven the large MDR-TB epidemics in the region. Notably, high transmissibility was attributed to the CAO clade, despite the accumulation of multiple resistance mutations that, theoretically, should have affected the bacterial fitness of Mtb strains.

To check the population structure of Mtb from the current sample, we mapped the WGS data onto the phylogenetic tree (Figure 2). As a result, the dominant transmission clusters of the isolates from Astana were assigned to the CAO clade of L2/Beijing (n = 15; 62.5%) (Table 2). Other clusters in the sample from Astana were represented by the CA clade (n = 5; 20.8%), Europe/Russia W148 outbreak (n = 3; 12.5%), and Clade A (n = 1; 4.2%). The CAO isolates were primarily characterized as pre-XDR (11/15) and XDR (1/15), exhibiting high levels of drug resistance. Conversely, the CA clade, represented by five isolates, was not associated with pre-XDR-TB or XDR-TB. Furthermore, three out of five CA isolates were susceptible to RIF and lacked non-synonymous mutations in *rpoABC* genes.

But what causes the CAO transmission cluster to be a driver of MDR-TB epidemics, making it a particular concern for public health in the former Soviet Union countries? One possible reason is the acquisition of compensatory mutations after the introduction of resistance mutations. Initially, Gagneux et al. reported that mutations associated with drug resistance impact the bacterial fitness of Mtb strains [17,38]. The resistant mycobacteria acquire additional mutations over time to compensate for the resulting reduction in fitness, reducing the initial fitness defects [39,40,41]. Notably, the acquisition of compensatory mutations after the introduction of mutations that confer resistance (e.g., *rpoB* S450L) is critical for higher transmission rates.

The occurrence of compensatory mutations strongly associated with the *rpoB* S450L mutation was described by Song et al.; compensatory variants were found in 50 (54%) of the 92 strains harboring S450L, compared to only 8 (10%) of the 78 isolates carrying other mutations in the *rpoB* gene (*p* < 0.0001) [42]. Other studies suggest a strong association between the RIF resistance-associated mutation S450L with compensatory mutations in *rpoABC* genes [38,43,44,45,46,47,48]. The association between compensatory mutations in *rpoABC* and *rpoB* S450L mutations has been well established by various experiments, including the generation of mutant Mtb clones, measurement of the growth rate of the resistant strain compared to the drug-sensitive parent, and their relative ability to cause TB disease or death in an animal model [49,50,51,52,53,54,55].

We then tried to gain further insights into the emergence of compensatory mutations in the CAO cluster, which dominate the Mtb population of Uzbekistan, and likely Kazakhstan. Merker et al. traced it back to the most recent common ancestor (MRCA) of the CAO clade, with an estimated mean age dating to sometime between 1966 and 1982. The Uzbekistani CAO isolates have subsequently acquired multiple mutations conferring resistance, as well as compensatory mutations. Particularly, two compensatory mutations, *rpoB* I488V and *rpoC* N698S, are deeply rooted in the CAO phylogeny. For example, mutation *rpoC* N698S accounts for 79/124 (63.7%) of CAO isolates circulating in Uzbekistan. These mutations have been associated with increased fitness in RR strains, leading to the observed transmission success and higher drug resistance within the CAO cluster in Uzbekistan. The mean age of the subclade with *rpoC* N698S was estimated to be dated to sometime between 1987 and 1994. Both compensatory mutations were present in 1998, when Uzbekistan implemented a DOTS strategy without fully understanding the true extent of the MDR-TB outbreak at that time [56].

Importantly, *rpoB* I488V and *rpoC* N698S compensatory mutations were not observed in the current study or other local studies. Nevertheless, isolates #9053-05 and #688-06 from Uzbekistan, which harbor the *rpoB* I488V and *rpoC* N698S mutations, cluster together on the phylogenetic tree with several isolates from Astana that do not have those compensatory mutations (Figure 2). It can be inferred that the local CAO clade likely diverged from the CAO clade in neighboring Uzbekistan before acquiring the *rpoB* I488V and *rpoC* N698S mutations. Therefore, the divergence of the two CAO subgroups may have occurred in the mid-1980s, before the collapse of the Soviet Union, when Kazakhstan and Uzbekistan constituted a single country without distinct borders. A total of 13 putative compensatory mutations in *rpoABC* genes were detected in the studied L2/Beijing isolates. All putative compensatory mutations were observed exclusively in the local RR-resistant isolates containing the *rpoB* S450L mutation. The most frequent putative compensatory mutation detected in four CAO clade isolates was *rpoC* V1039A.

To gain insights into the effects of the observed putative compensatory mutations on *rpoA*, *rpoB*, and *rpoC* proteins, we assessed the impact on the stability and flexibility of appropriate protein structures in silico. Based on the calculated changes in Gibbs free energy affecting protein stability, the acquisition of compensatory mutations had a stabilizing effect on the RNAP subunits encoded by *rpoA*, *rpoB,* and *rpoC*, except for the *rpoC* V483G mutation, which showed negative predicted stability changes, that could potentially lead to a destabilizing effect (Table 4). Song et al. described the involvement of this specific compensatory mutation in the restoration of RNAP activity [42]. According to the ML tree (Figure 2), the distinct *rpoC* V483G mutation was acquired independently multiple times, as it appears in phylogenetically separate branches, suggesting possible convergent compensation for the *rpoB* S450L mutation. Another example of potential convergent compensation for *rpoB* S450L is the *rpoC* G519S mutation, which was detected in phylogenetically separate CAO and CA clades. Notably, *rpoC* V483G and G519S are located in the same region as the exit tunnel in the beta’ subunit (Figure S2b). As reported by Song et al., the RIF binding pocket in RNAP runs along the exit tunnel, where newly synthesized RNA is formed at the interface of the beta and beta’ subunits [42]. The putative compensatory mutations in the current study (V483G, D485N, V517L, and G519S) are located in the alpha helices at the end of the exit tunnel in the beta’ subunit. The convergent evolution of these mutations in the same region underscores their importance in the compensatory function linked to the S450L RR conferring mutation.

## 4. Conclusions

In conclusion, the high prevalence of the CAO clade in the population structure of Mtb may explain the rapid spread of MDR-TB in Kazakhstan, as follows from the recent expansion of DR isolates in Uzbekistan, linked to the spread of the “successful” CAO sublineage of the Modern Beijing genotype [22]. The CAO clade originated in the mid-1970s in the Uzbek Republic of the former Soviet Union, and most likely diverged right before the collapse of the Soviet Union. After the divergence, *rpoB* I488V and *rpoC* N698S compensatory mutations emerged in the Mtb population from Uzbekistan, while other putative compensatory mutations emerged in the locally circulating transmission clusters. Upon the collapse of the Soviet Union, the situation with MDR-TB was worsened by inadequate TB diagnosis and treatment practices, limited access to appropriate drugs, and insufficient infection control measures in healthcare settings [25,57,58]. Although most of these problems have now been solved in Kazakhstan, we continue to observe the consequences of these 30-year-old circumstances.

To address the MDR-TB outbreak, the Kazakhstan government has taken several measures. These include strengthening the National TB Control Program, forging partnerships with organizations such as the Global Fund and the World Health Organization, and implementing strategies to improve TB diagnosis, treatment, and prevention in order to reduce the spread of the disease [56,59]. Despite these commendable efforts, the drug-resistant TB (DR-TB) situation in Kazakhstan continues to be challenging. Further research is necessary to enhance molecular diagnostics, investigate outbreaks more effectively, and improve the management of DR-TB in the country. Our future work should include exploring variants with potential phenotypic compensatory effects, co-selected with mutations conferring resistance to BDQ, and novel anti-TB drugs. Another future direction is to study a country-wide representative sample of L2/Beijing isolates collected from all regions of Kazakhstan and to employ Bayesian phylogenetic analysis for CAO transmission cluster and mutation rate estimation. This would provide a more accurate picture of ongoing Mtb transmission and evolution processes in the region.

## 5. Materials and Methods

### 5.1. Study Population and Drug Susceptibility Testing

Convenience sampling was used to collect 29 clinical isolates cultured from the sputum of patients with clinically suspected TB at Astana City Center for Phthisiopulmonology (Astana, Kazakhstan) in 2021–2022. The study was approved by the Ethics Committee of the National Center for Biotechnology under the Ministry of Health of the Republic of Kazakhstan (Protocol #3, approved 7 August 2020). The drug susceptibility of all Mtb cultures was tested using a Bactec MGIT 960 culture system (Becton Dickinson, Cockeysville, USA) according to the manufacturer’s protocol for first-line (isoniazid (INH), rifampicin (RIF), streptomycin (STR), ethambutol (EMB), and pyrazinamide (PZA)) and second-line (levofloxacin (LVX), moxifloxacin (MFX), amikacin (AMK), kanamycin (KAN), capreomycin (CAP), bedaquiline (BDQ), and clofazimine (CFZ)) drugs. The critical concentrations of anti-tuberculosis drugs used for drug susceptibility were predefined by the Ministry of Health of the Republic of Kazakhstan (Table S3). The anti-TB drugs and dosages used in the country are represented in Tables S4 and S5. The studied sample was represented by susceptible (sensitive), other (not RIF-resistant), MDR, Pre-XDR, and XDR isolates.

### 5.2. DNA Isolation and Genotyping

DNA was extracted using the cetyltrimethylammonium (CTAB) procedure [60]. A total of 29 Mtb isolates were genotyped using a real-time PCR assay targeting the *dnaA-dnaN*::IS*6110* region specific to L2/Beijing isolates, as described by Mokrousov et al. [18]. Five non-Beijing isolates were excluded from the sample based on the genotyping results. The quality of the DNA was checked using a Qubit dsDNA Quantification Assay Kit (Thermo, Eugene, OR, USA) and a Qubit 2.0 fluorometer (Thermo, Carlsbad, CA, USA).

### 5.3. Whole-Genome Sequencing

A total of 24 L2/Beijing isolates were sequenced using a MiSeq platform (Illumina, San Diego, CA, USA). For the MiSeq sequencing, libraries with an average fragment size of 600 bp were prepared using a DNA Prep kit (Illumina, San Diego, CA, USA) according to the manufacturer’s instructions. FASTQ files were deposited onto the NCBI Sequence Read Archive (see Table S1 for accession numbers).

The reads from all Mtb genomes were mapped to the *M. tuberculosis* H37Rv genome (GenBank ID: NC_000962.3) using the MTBseq pipeline v.1.0.3 [61]. Variants (SNPs and InDels) were called if the following criteria were met: minimum coverage of four reads per direction (forward and reverse), minimum Phred score of 20, and allele frequency of at least 75%.

PhyResSe and TB-Profiler online tools were used to check for antibiotic resistance and heteroresistance in the obtained WGS data [20,62]. The annotation of genomes was performed using the NCBI Prokaryotic Genome Annotation Pipeline [63]. In silico spoligotyping was performed using SpoTyping 2.1, and the assignment of regions of deletion was accomplished using TB-Profiler [19,20].

### 5.4. Prediction of Protein Stability and Dynamics

Dynamut was used to predict the effects of single point mutations on protein stability (changes in folding free energy between wild type and mutant, ΔΔG (kcal/mol)) and dynamics (changes in vibrational entropy energy between wild type and mutant, ΔΔS_Vib_ (kcal/mol)) of *M. tuberculosis* H37Rv RNAP subunits encoded by *rpoA*, *rpoB*, and *rpoC*. Default parameters were used unless otherwise specified. The *M. tuberculosis* RNAP model was downloaded from PDB (PDB ID: 6c04) [64].

### 5.5. Phylogenetic Analysis

For phylogenetic analysis, a concatenated SNP alignment was created based on variant positions, excluding insertions and deletions, SNPs located within 12 bp or in repetitive regions, or resistance-associated genes using MTBseq pipeline v.1.0.3, as described elsewhere [61]. This pipeline employs the widely used open-source programs BWA, SAMtools, PICARD-tools and Genome Analysis Toolkit (GATK) [65,66,67]. An SNP alignment was created from a total of 47 MTBC whole-genome sequences (Table S1), including 24 sequences from the current study, 7 sequences of L2/Beijing isolates published by Merker et al., and 16 reference sequences of MTBC clinical strains published by Borrell et al. [25,26]. The obtained high-confidence SNPs were written to a multi-FASTA alignment.

The multi-FASTA alignment was used to calculate the maximum likelihood tree with FastTree v.2.1.11 using the general time reversible (GTR) model of nucleotide substitution, 1000 resamplings, and Gamma20 likelihood optimization to account for evolutionary rate heterogeneity among sites [68]. The consensus tree was rooted with the “midpoint root” option in FigTree 1.4.4, and nodes were arranged in an increasing order [69]. MTBC strains were stratified into lineages and subgroups using the classification schemes by Coll et al. and Merker et al. [21,22]. A pairwise SNP distance matrix was calculated using MTBseq v.1.0.3 (Table S2).

## Figures and Tables

**Table 1:** List of 24 Mtb isolates from Astana, Kazakhstan, with confirmed L2/Beijing genotype, diagnosis, demographic data, and type of phenotypic drug resistance.

Isolate	Gender	Age	DR Type	Diagnosis (TB)	Case
1028	Female	35	Pre-XDR	Infiltrative pulmonary	New
1561	Male	42	Pre-XDR	Infiltrative pulmonary	Relapse
3775	Male	44	Pre-XDR	Miliary	New
4800	Male	40	Pre-XDR	Infiltrative pulmonary	Retreatment
5023	Female	34	Sens	Infiltrative pulmonary	New
5099	Female	60	Pre-XDR	Infiltrative pulmonary	Relapse
5135	Male	29	Pre-XDR	Infiltrative pulmonary	New
5194	Female	30	MDR	Infiltrative pulmonary	New
5195	Male	25	Pre-XDR	Infiltrative pulmonary	New
5219	Female	60	Pre-XDR	Infiltrative pulmonary	Relapse
5264	Male	41	Pre-XDR	Fibrocystic cavernous	Relapse
5313	Male	31	MDR	Infiltrative pulmonary	New
5382	Female	61	MDR	Infiltrative pulmonary	New
5628	Male	47	Pre-XDR	Infiltrative pulmonary	Relapse
6242	Female	44	Other (S)	Infiltrative pulmonary	Relapse
6466	Female	52	MDR	Infiltrative pulmonary	Relapse
6616	Male	44	Pre-XDR	Miliary	New
6646	Male	50	Pre-XDR	Infiltrative pulmonary	Retreatment
7076	Male	39	Sens	Infiltrative pulmonary	New
7357	Male	54	MDR	Fibrocystic cavernous	Relapse
7389	Male	54	Pre-XDR	Infiltrative pulmonary	Relapse
7678	Male	37	Pre-XDR	Infiltrative pulmonary	New
7923	Female	39	XDR	Infiltrative pulmonary	New
8316	Female	49	Pre-XDR	Infiltrative pulmonary	New

**Table 2:** Spoligotype, region of difference (RD), and sublineage classification of 24 Mtb isolates from Astana, based on WGS data.

Isolate	Spoligotype	RD	Sublineage [21]	Sublineage [22]	Sublineage [23]
1028	000000000003771	RD105, RD207, RD181	2.2.1	Central Asia outbreak	L2.2.M4.9.1
1561	000000000003771	RD105, RD207, RD181	2.2.1	Europe/Russian W148 outbreak	L2.2.M4.5
3775	000000000003771	RD105, RD207, RD181	2.2.1	Central Asia outbreak	L2.2.M4.9.1
4800	000000000003771	RD105, RD207, RD181	2.2.1	Central Asia outbreak	L2.2.M4.9.1
5023	000000000003771	RD105, RD207, RD181	2.2.1	Central Asia	L2.2.M4.9
5099	000000000003771	RD105; RD207; RD181	2.2.1	Central Asia outbreak	L2.2.M4.9.1
5135	000000000003771	RD105; RD207; RD181	2.2.1	Central Asia outbreak	L2.2.M4.9.1
5194	000000000003771	RD105; RD207; RD181	2.2.1	Central Asia outbreak	L2.2.M4.9.1
5195	000000000003771	RD105; RD207; RD181	2.2.1	Central Asia outbreak	L2.2.M4.9.1
5219	000000000003771	RD105; RD207; RD181	2.2.1	Central Asia outbreak	L2.2.M4.9.1
5264	000000000003771	RD105; RD207; RD181	2.2.1	Europe/Russian W148 outbreak	L2.2.M4.5
5313	000000000003771	RD105; RD207; RD181	2.2.1	Central Asia outbreak	L2.2.M4.9.1
5382	000000000003771	RD105; RD207; RD181	2.2.1	Central Asia	L2.2.M4.9
5628	000000000003771	RD105; RD207; RD181	2.2.1	Europe/Russian W148 outbreak	L2.2.M4.5
6242	000000000003771	RD105; RD207; RD181	2.2.1	Central Asia	L2.2.M4.9
6466	000000000003771	RD105; RD207; RD181	2.2.1	Central Asia	L2.2.M4.9
6616	000000000003771	RD105; RD207; RD181	2.2.1	Central Asia outbreak	L2.2.M4.9.1
6646	000000000003771	RD105; RD207; RD181	2.2.1	Central Asia outbreak	L2.2.M4.9.1
7076	000000000003771	RD105; RD207; RD181	2.2.1	Central Asia	L2.2.M4.9
7357	000000000003771	RD105; RD207; RD181	2.2.1	Central Asia outbreak	L2.2.M4.9.1
7389	000000000003771	RD105; RD207; RD181	2.2.1	Central Asia outbreak	L2.2.M4.9.1
7678	000000000003771	RD105; RD207; RD181	2.2.1	Central Asia outbreak	L2.2.M4.9.1
7923	000000000003771	RD105; RD207; RD181	2.2.1	Central Asia outbreak	L2.2.M4.9.1
8316	000000000003771	RD105; RD207; RD181	2.2.1	Clade A	L2.2.M4.9.2

**Table 3:** Drug resistance profiles of 24 Mtb isolates from Astana, based on WGS data. Boldface indicates discrepancy with phenotypic resistance.

Drug	INH	RIF	EMB	STM	PZA	OFX	KAN	AMK	CAP	ETO	BDQ
Isolate											
1028	R	R	R	R	S	R	S	S	S	S	S
1561	R	R	R	R	R	R	S	S	S	S	R
3775	R	R	R	R	S	R	S	S	S	S	S
4800	R	R	R	R	R	R	R	R	R	S	S
5023	S	S	S	S	S	S	S	S	S	S	S
5099	R	R	R	R	S	R	S	S	S	S	S
5135	R	R	R	R	S	R	S	S	S	S	S
5194	R	R	R	R	R	S	S	S	S	S	S
5195	R	R	R	R	R	R	S	S	S	S	S
5219	R	R	R	R	S	R	S	S	S	S	S
5264	R	R	R	R	R	R	S	S	S	S	R
5313	R	R	R	R	S	S	S	S	S	S	S
5382	R	R	R	R	R	S	R	R	R	S	S
5628	R	R	R	R	R	R	R	S	S	S	S
6242	S	S	S	R	S	S	S	S	S	S	S
6466	R	R	R	R	S	S	S	S	S	R	S
6616	R	R	R	R	S	R	S	S	S	S	S
6646	R	R	R	R	S	R	S	S	R	S	S
7076	S	S	S	S	S	S	S	S	S	S	S
7357	R	R	R	R	R	S	S	S	S	S	S
7389	R	R	R	R	R	R	S	S	S	S	R
7678	R	R	R	R	S	R	S	S	S	S	S
7923	R	R	R	R	S	R	S	S	S	S	R
8316	R	R	R	S	S	R	R	S	S	R	S

INH, isoniazid; RIF, rifampicin; EMB, ethambutol; STM, streptomycin; PZA, pyrazinamide; OFX, ofloxacin; KAN, kanamycin; AMK, amikacin; CAP, capreomycin; ETO, ethionamide; BDQ, bedaquiline.

**Table 4:** Compensatory variants of RR-conferring *rpoB* S450L mutation. Boldface indicates previously unreported variant.

Gene	Compensatory Mutation	Frequency	P/NP	HP/HB	Energy (ΔΔG, kcal/mol)	Flexibility (ΔΔS_Vib_, kcal/mol)
*rpoC*	V1039A	4	NP-NP	HB-HB	1.266	−3.570
*rpoC*	D485N	2	P-P	HP-HP	0.917	−4.029
*rpoC*	G519S	2	NP-P	HB-HP	1.024	−3.801
*rpoC*	K717Q	2	P-P	HP-HP	1.028	−3.533
*rpoA*	T187A	2	P-NP	HP-HB	0.143	0.104
*rpoC*	V431M	1	NP-NP	HB-HB	1.064	−4.096
*rpoC*	V483G	1	NP-NP	HB-HB	−0.793	−3.064
*rpoC*	V517L	1	NP-NP	HB-HB	1.785	−4.121
*rpoB*	Y564H	1	P-P	HP-HP	1.399	−3.783
*rpoB*	T585S	1	P-P	HP-HP	0.025	−4.287
*rpoB*	E761D	1	P-P	HP-HP	1.463	−4.275
*rpoA*	D190A	1	P-NP	HP-HB	0.025	0.595
*rpoA*	G31S	1	NP-P	HB-HP	0.083	−0.448

P, polar; NP, non-polar; HP, hydrophilic; HB, hydrophobic; S_Vib_, vibrational entropy.

## Data Availability

The raw data from BioProject PRJNA980215 were submitted to the NCBI Sequence Read Archive (see Table S1 for accession numbers).

## Supplementary Materials

The following supporting information can be downloaded at https://www.mdpi.com/article/10.3390/antibiotics12101523/s1. Table S1. Metadata, phenotypic drug susceptibility testing results, compensatory mutations and classification for the studied clinical Mtb isolates; Table S2. Pairwise SNP distance matrix of 47 MTBC genomic DNA sequences; Table S3. Critical concentrations of anti-tuberculosis drugs used for drug susceptibility testing; Table S4. Daily doses (mg) of anti-tuberculosis drugs for the treatment of drug-susceptible tuberculosis in Kazakhstan; Table S5. Daily doses (mg) of anti-tuberculosis drugs for the treatment of drug-resistant tuberculosis in Kazakhstan. Figure S1. Fluorescence curves of a real-time PCR assay targeting the *dnaA-dnaN*::IS*6110* region of *M. tuberculosis* L2/Beijing genotype; Figure S2. Three-dimensional representation of compensatory mutations on the crystal structure of Mtb DNA-directed RNA polymerase (RNAP) subunit beta.
