# Supplementary material for: Genomic Characterization of Drug-Resistant Mycobacterium tuberculosis L2/Beijing Isolates from Astana, Kazakhstan
Source: Antibiotics (Basel). 2023 Oct 10;12(10):1523. doi: 10.3390/antibiotics12101523 (PMC10604462; doi:10.3390/antibiotics12101523)
Supplement: Supplementary file 1 [file antibiotics-12-01523-s001.zip › Figures S1 and S2.pdf]

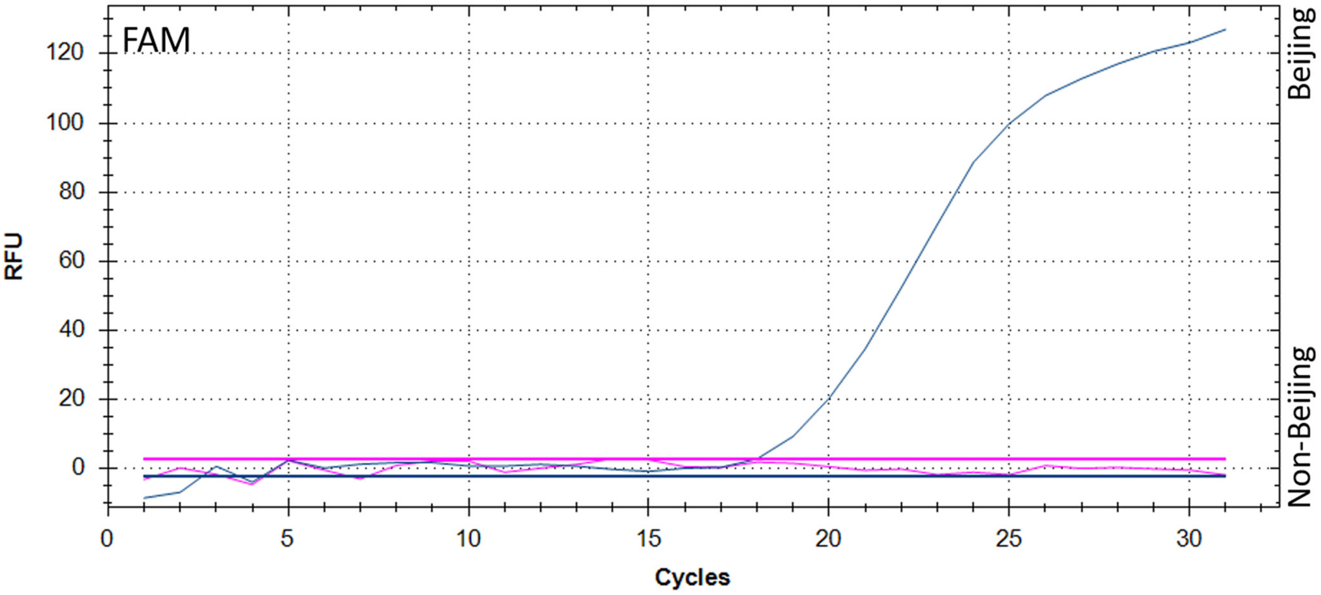

(**a**)

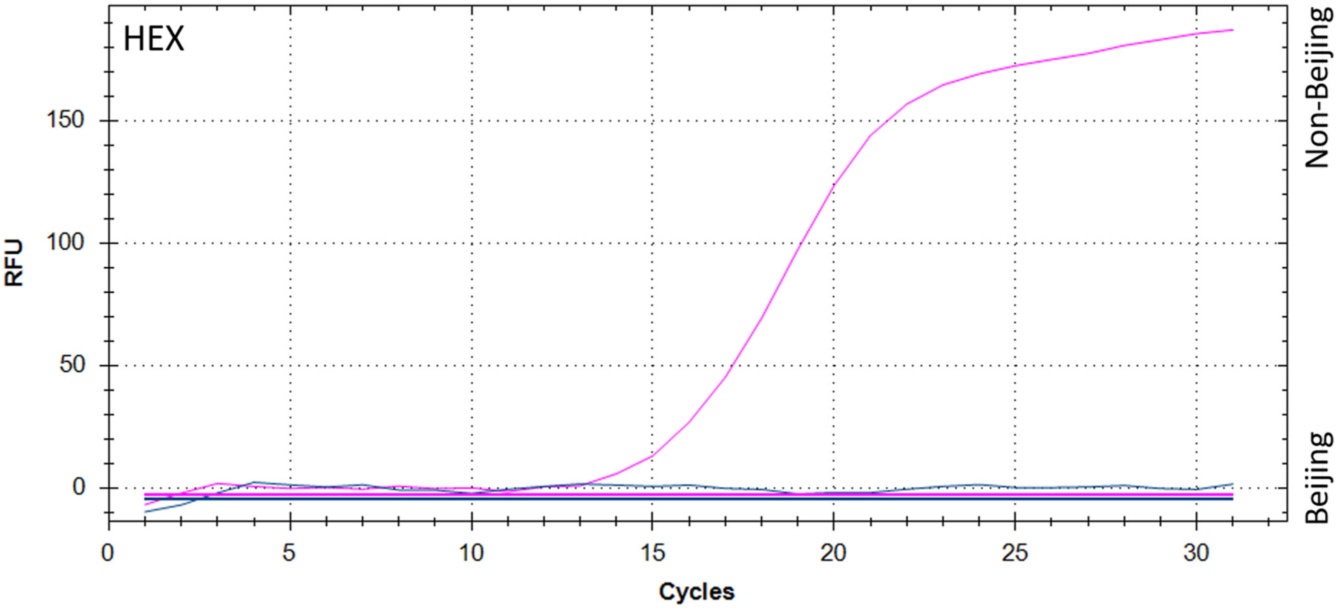

(**b**)

Figure S1. Fluorescence curves of a real-time PCR assay targeting the *dnaA-dnaN*::IS*6110* region of *M. tuberculosis* L2/Beijing genotype: (**a**) L2/Beijing genotype-specific signal (FAM channel); (**b**) Non-Beijing genotype-specific signal (HEX channel). The design of the PCR assay was described by Mokrousov et al. [18].

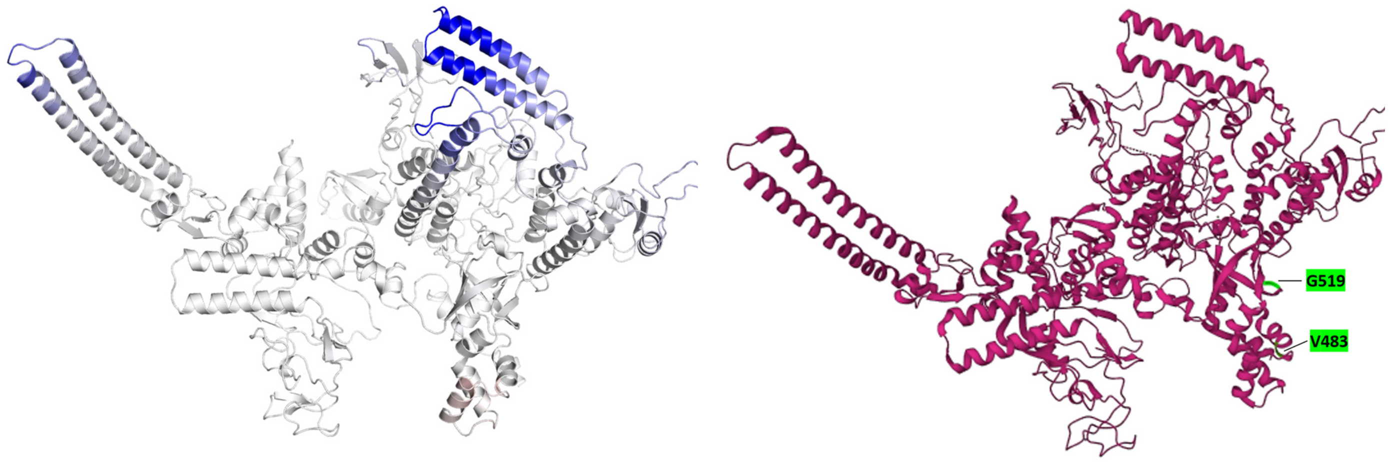

(**a**) (**b**)

Figure S2. Three-dimensional representation of compensatory mutations on the crystal structure of Mtb DNA-directed RNA polymerase (RNAP) subunit beta (PDB: 6c04). (**a**) Decreased flexibility of RNAP subunit beta upon V483G mutation. Amino acids are colored according to vibrational entropy change (blue: rigidification of structure; red: gain in flexibility). (**b**) RNAP subunit beta structure with V483 and G519 WT alleles (green).
